# Innovations in Promoting and Advancing Age-Friendly Principles After Achieving AFU Designation

**DOI:** 10.1093/geroni/igaf122.1931

**Published:** 2025-12-31

**Authors:** Katarina Friberg-Felsted, Jacqueline Eaton, M Aaron Guest

## Abstract

Receipt of the Age-Friendly University designation from the AFU Global Network (AFUGN) allows institutions to further progress in the development of age-friendly practices in a variety of academic and community settings. Institutional representatives from various parts of the world describe the unique ways they have advanced age-friendly and age-inclusive contributions and share concrete ways forward. Themes include multifaceted strategies and activities to increase visibility on campus and in the community, as well as ways to foster an inclusive and supportive environment for all ages. Peters-Beumer will describe the age-friendly/age-inclusive processes undertaken by Concordia University’s Center for Gerontology, including facilitating programs with community partners (such as Reframing Aging); Doron will outline how the University of Haifa’s eight impactful initiatives align with the domains of the Age Inclusivity Domains of Higher Education (AIDHE) model; Elfenbein will explore how AFU status led to the creation of the University of North Georgia Institute for Healthy Aging; Shovali will share Eckerd College’s multifaceted approach to raise awareness and highlight the value of working in an age-diverse environment; and Waterhouse will showcase age-friendly practical strategies and specific projects successfully executed by the University of Colorado Anschutz Medical Campus. A discussion focusing on advancing and AFU initiatives will be led by AFUGN Secretariat Chair, Aaron Guest.

